# Diurnal Variations of Circulating Extracellular Vesicles Measured by Nano Flow Cytometry

**DOI:** 10.1371/journal.pone.0144678

**Published:** 2016-01-08

**Authors:** Kirsty M. Danielson, Jessica Estanislau, John Tigges, Vasilis Toxavidis, Virginia Camacho, Edward J. Felton, Joseph Khoory, Simion Kreimer, Alexander R. Ivanov, Pierre-Yves Mantel, Jennifer Jones, Praveen Akuthota, Saumya Das, Ionita Ghiran

**Affiliations:** 1 Department of Medicine, Beth Israel Deaconess Medical Center and Harvard Medical School, Boston, MA, United States of America; 2 Barnett Institute of Chemical and Biological Analysis, Northeastern University, Boston, MA, United States of America; 3 Department of Chemistry and Chemical Biology, Northeastern University, Boston, MA, United States of America; 4 Molecular Immunogenetics & Vaccine Research Section Vaccine Branch, CCR, NCI, Bethesda, MD, United States of America; European Institute of Oncology, ITALY

## Abstract

The identification of extracellular vesicles (EVs) as intercellular conveyors of biological information has recently emerged as a novel paradigm in signaling, leading to the exploitation of EVs and their contents as biomarkers of various diseases. However, whether there are diurnal variations in the size, number, and tissue of origin of blood EVs is currently not known, and could have significant implications when using EVs as biomarkers for disease progression. Currently available technologies for the measurement of EV size and number are either time consuming, require specialized equipment, or lack sufficient accuracy across a range of EV sizes. Flow cytometry represents an attractive alternative to these methods; however, traditional flow cytometers are only capable of measuring particles down to 500 nm, which is significantly larger than the average and median sizes of plasma EVs. Utilizing a Beckman Coulter MoFlo XDP flow cytometer with NanoView module, we employed nanoscale flow cytometry (termed nanoFCM) to examine the relative number and scatter distribution of plasma EVs at three different time points during the day in 6 healthy adults. Analysis of liposomes and plasma EVs proved that nanoFCM is capable of detecting biologically-relevant vesicles down to 100 nm in size. With this high resolution configuration, we observed variations in the relative size (FSC/SSC distributions) and concentration (proportions) of EVs in healthy adult plasma across the course of a day, suggesting that there are diurnal variations in the number and size distribution of circulating EV populations. The use of nanoFCM provides a valuable tool for the study of EVs in both health and disease; however, additional refinement of nanoscale flow cytometric methods is needed for use of these instruments for quantitative particle counting and sizing. Furthermore, larger scale studies are necessary to more clearly define the diurnal variations in circulating EVs, and thus further inform their use as biomarkers for disease.

## Introduction

The ability to detect diseases early in the pre-clinical stage with high sensitivity and specificity through minimally invasive methods is essential for more effective intervention and improved clinical outcomes. In the past decade, extracellular vesicles (EVs) have been identified in body fluids and have gained prominence as novel cell-cell communicators as well as biomarkers for prognosis and diagnosis of disease. EVs are a heterogeneous group of vesicles that include exosomes [1], microvesicles [2], and apoptotic bodies [3]. Particular attention has been paid to EVs as biomarkers in blood, where they are postulated to be involved in numerous pathological and physiological processes. They have been reported to carry cellular components that may have functional effects on neighboring or distant cells including mRNA and microRNA [4], other non-coding RNA [5], cytoplasmic and membrane proteins [6], and lipids [7]. Little is known about normal EV physiology, regulation of production, and release into biological fluids. As a result, current experimental designs may fail to consider important factors that influence the production and regulation of circulating EVs.

Diurnal variations in circulating factors result from numerous influences, including: diet, exercise, and circadian variations that are characterized by regular oscillations in physiological parameters within a period of approximately 24 hours [8]. The functional consequences of these variations and cycles are critical in the interactions between the central nervous, endocrine, and immune systems [9, 10]. During the day/night cycle, the oscillation of blood hormone levels such as growth hormone (GH), prolactin, cortisol, and catecholamines regulate cytokine production, leukocyte trafficking and activation, and T-cell proliferation and differentiation [11]. Given the impact of diurnal variations on numerous blood-borne factors and the increasing interest in circulating blood EVs as biomarkers of disease, the temporal variation in size and number of blood EVs is an area that warrants investigation.

Nanoscale flow cytometry (termed nanoFCM) is a novel technology that has the advantage of identifying particles as small as 100 nm in diameter. Importantly, the processing of plasma for flow cytometry-based analysis has a minimal impact on EV size and distribution, thus the results are likely to be representative of the number and size-distribution of EVs in blood. Here we show that this technology is capable of detecting EVs down to 100 nm in size, and our results suggest that the relative number and size distribution of EVs in the plasma of healthy donors changes during the day.

## Materials and Methods

### Subjects

Blood was obtained from healthy adult volunteers in accordance with the guidelines of, and approved by the Institutional Review Board of Beth Israel Deaconess Medical Center. Donors from multiple diversities were recruited by local advertising and written informed consent was obtained from each volunteer in accordance with the Declaration of Helsinki. Approved study staff was responsible for all blood collection, processing, and documentation of consent files. Blood was obtained from 6 healthy adult volunteers with no control over diet and exercise throughout the day in order to better mimic regular daily fluctuations in individuals.

### Preparation of Plasma and Isolation of EVs

Donors from multiple diversities were recruited by local advertising and written informed consent was obtained from each volunteer in accordance with the Declaration of Helsinki. Approved study staff was responsible for all blood collection, processing, and documentation of consent files. Blood (5 mL) was obtained by percutaneous cubital venipuncture drawn in an EDTA containing vacutainer (BD Biosciences, Franklin Lakes, NJ) at 3 time points (7.30 am, 2.30 pm, 7.30 pm; donor 4 did not provide final sample) using a gauge 19 needle (BD Biosciences). None of the donors necessitated the use of a tourniquet. Blood was centrifuged at 500 x g for 5 min to obtain plasma, followed by two plasma centrifugations; 5,000 x *g* for 15 min and 12,000 x g for 15 minutes at room temperature. Plasma was centrifuged at room temperature to prevent activation of platelets and the release of platelet-derived EVs; Samples were then processed in microcentrifuge tubes that showed no significant ‘shed’ (USA Scientific, 1415–2500) of debris recorded by the instrument in the size range of EVs and indistinguishable from EVs (S1 Fig). The lipid-rich topmost layer (500–700 μL) was discarded and 10 μL of the plasma was diluted 1:500 in Phosphate Buffered Saline (PBS; Life Technologies, Carlsbad, CA), which was previously pre-filtered through a 0.2 μm filter. Similarly, the sheet fluid from the flow cytometer was filtered through a built-in 0.2 μm filter. All samples were analyzed by nanoFCM within 30 minutes post processing and all procedures were performed at room temperature.

### Liposome Preparation

Phospholipid vesicles comprised of a molar fraction of 50% synthetic 1,2-dioleoyl-sn-glycero-3-phosphocholine (DOPC), 30% chicken egg L-α-phosphatidylcholine (PC), and 20% porcine brain L-α- phosphotidylserine (PS) (Avanti Polar Lipids; Alabaster, AL) were prepared according to the methods of Shi et al., 2004 [12]. Phospholipids dissolved in chloroform were obtained from Avanti Polar Lipids (Alabaster, AL). 1.3 μmol DOPC, 0.78 μmol PC, and 0.52 μmol PS were added to dichloromethane (99.8%, Acros Organics, Morris Plains, NJ) in an acid-cleaned glass vial, and the solvent was evaporated under a gentle nitrogen stream while immersed in a 60°C water bath. Additional dichloromethane was added and evaporated from the vial three times to eliminate traces of chloroform. 1 mL of HBS (100 mMNaCl, 20mM HEPES, 0.02% w/v sodium azide, pH 7.5) was added to the dried phospholipids and vortexed until all phospholipids were dissolved, resulting in a uniform, cloudy solution. Liposomes were formed by extruding this solution through a double layer of polycarbonate membranes with 100 nm pore size (Nuclepore Track-Etch, Whatman, Maidstone, UK) 21 times using the Mini-Extruder extrusion device (Avanti Polar Lipids).

### Instrumentation

The NanoView module (Propel Labs, Fort Collins, CO) replaces the traditional Forward Scatter Photodiode Detector (FSC) on a Beckman Coulter MoFlo XDP cell sorter in order to extend the FSC detection resolution below the traditional 500 nm lower limit. The NanoView system includes several custom designed components aimed at maximizing the relevant event-generating signal, and reducing the noise in the forward scatter collection path. The first significant difference is a modified nozzle chamber located between the interrogation point and the FSC collection optics, which increases the vertical opening, allowing more light and larger angles of the scattered light, up to 18° or twice the amount of the standard MoFlo XDP, to be collected by the FSC lens. The FSC lens in the NanoView module is an aspheric element that focuses the light scattered by the particles from the interrogation point in the stream through a 200 μm pinhole, thus rejecting the scattered light originating from out-of-focus areas. A Hamamatsu (Boston, MA) PMT detector (C6270No.AA4834) is used to further increase the signal strength from a 200 mW Coherent Sapphire 488 nm solid state laser. The final design element is an adjustable scatter bar, which allows optimizing the angles of scattered light collected and blocked. This enables the MoFlo XDP system to be used for smaller or larger particles based on the blocking angle adjustment and PMT gain settings (S2 Fig). A standard LSRII instrument (20mW Coherent Sapphire solid state laser with FSC photodiode and SSC PMT in the optical Octagon configuration) was used for comparison against the nanoFCM. Samples were acquired on a medium pressure setting and a SSC threshold of 200 (the minimal threshold allowed on a LSR II). Noise was calculated by acquiring a PBS alone sample and a gate was placed accordingly. Both 200 nm and 500 nm Photon Correlation Spectroscopy (PCS) controls (see section below) were acquired separately to set gates. The samples were mixed for verification of both populations. In both the 200 nm alone and mixed populations, 200 nm PCS controls were not separated from noise.

### NanoFCM Analysis

Photon Correlation Spectroscopy (PCS) latex beads (100 nm, 200 nm, 300 nm and 500 nm; PN 6602336, Beckman Coulter, Fullerton, CA), liposomes, and cell-free plasma were analyzed on the NanoView-equipped MoFlo XDP cell sorter by first optimizing the Coherent Sapphire 488–200 mW Laser at maximum output. A 70 μm nozzle tip was used and sheath pressure set to 60 PSI. Sheath fluid was filtered through a 0.2 μm Pall filter. Beckman Coulter Flow-Check Pro Flourospheres (cat # A69183) were used to locate bead spot and peak the 488 nm laser. This was accomplished by adjusting the micrometers for the laser stage and FSC stage. Next, adjustments were made to the FSC and SSC-related settings, voltage and threshold, (FSC log 425-475V, SSC log 400-450V, FSC or SSC threshold of 0.02%) to optimize the instrument for small particle detection. Both FSC and SSC threshold parameters were tested at the percentage threshold listed. Similar results were obtained for both scatter parameters; therefore, threshold parameter used was based on signal to noise ratio of samples being acquired.

Acquisition was started without any loaded sample by lowering the threshold initially to its minimum of 0.01 to visualize instrument noise generated by the drop drive, laser, and PMTs. The threshold was then raised to eliminate this noise population. Second, noise generated from filtered PBS alone was visualized, and a gate was drawn around this population (background noise). Finally, the experimental sample was measured and compared to the background noise. A second gate was drawn to above the initial gate and was inclusive of all events generated by the experimental sample. During analysis of all experimental samples, the gates were left the same for consistency and to allow for comparison of the GeoMeans.

In order to generate a size distribution curve, PCS controls were acquired in the following size ranges: 100 nm, 200 nm, 300 nm and 500 nm. Beginning with the 500 nm size bead population, followed by the remaining bead sizes down to the 100 nm bead sizes, adjustments were made to the FSC micrometers and the 488 nm laser micrometers to enhance dynamic range. For this study, the largest available blocker bar was used (±6.3°–±12.6° angles blocked as rotate from horizontal to vertical) and set to an approximate angle of 45° for best signal to noise ratio settings. The beads were mixed for a final bead size distribution curve. To prevent bead aggregation, microbeads were diluted in PBS with 0.1% Tween-20 solution, to a final concentration of 1.29x10^7^ beads/mL, sonicated, and centrifuged at 14,000 x g for 20 minutes to pellet bead aggregates. The topmost 10 μL were further diluted 1:100 before analysis. To further optimize for small particle detection, 100 nm liposomes were produced and analyzed as a size marker more closely resembling biological vesicles. All controls and samples were acquired at a pressure differential of 0.3–0.5 PSI. At this PSI setting and plasma dilution, EVs were processed at 10k to 20k events per second. For data analysis of EVs, the mean is chosen due to the scatter properties being logarithmic. The geometric mean (geomean) is chosen over the arithmetic mean due its robustness and not easily being affected by outliers. Therefore, to compare the populations of interest generated on the FSC Log vs SSC Log, the geomeans for both axes are compared. The change in geomean will reflect population shifts and allow for relative size comparisons.

### Atomic Force Microscopy

Atomic Force Microscopy was conducted at the Center for Nanoscale Systems at Harvard University (Cambridge, MA). Post-sorting, samples were cast onto a 10 mm mica plate for no more than 24 hours in a humidified environment. The samples were rinsed with deionized water to remove any crystallized ions and drops of water were added to the mica plate. Samples were then imaged using an Asylum-2 MFP-3D Coax Atomic Force Microscope and probed with a Biolever Mini cantilever set to AC Tapping mode in liquid. All images were analyzed using Asylum Research Analytical Tools.

### EV size analysis using tunable resistive pulse sensing

EV size analysis was performed using the qNano system (Izon) with a NP100 pore set to a fixed voltage and stretch. Size calibration was performed using 100 nm standard beads (Izon). Plasma EVs sorted into PBS were measured in 3 separate recordings with 40 μL additions to the chamber and a total of 535 individual EVs were counted and measured.

## Results

### NanoFCM allows identification of latex beads and liposomes down to 100 nm

**Fig 1:**
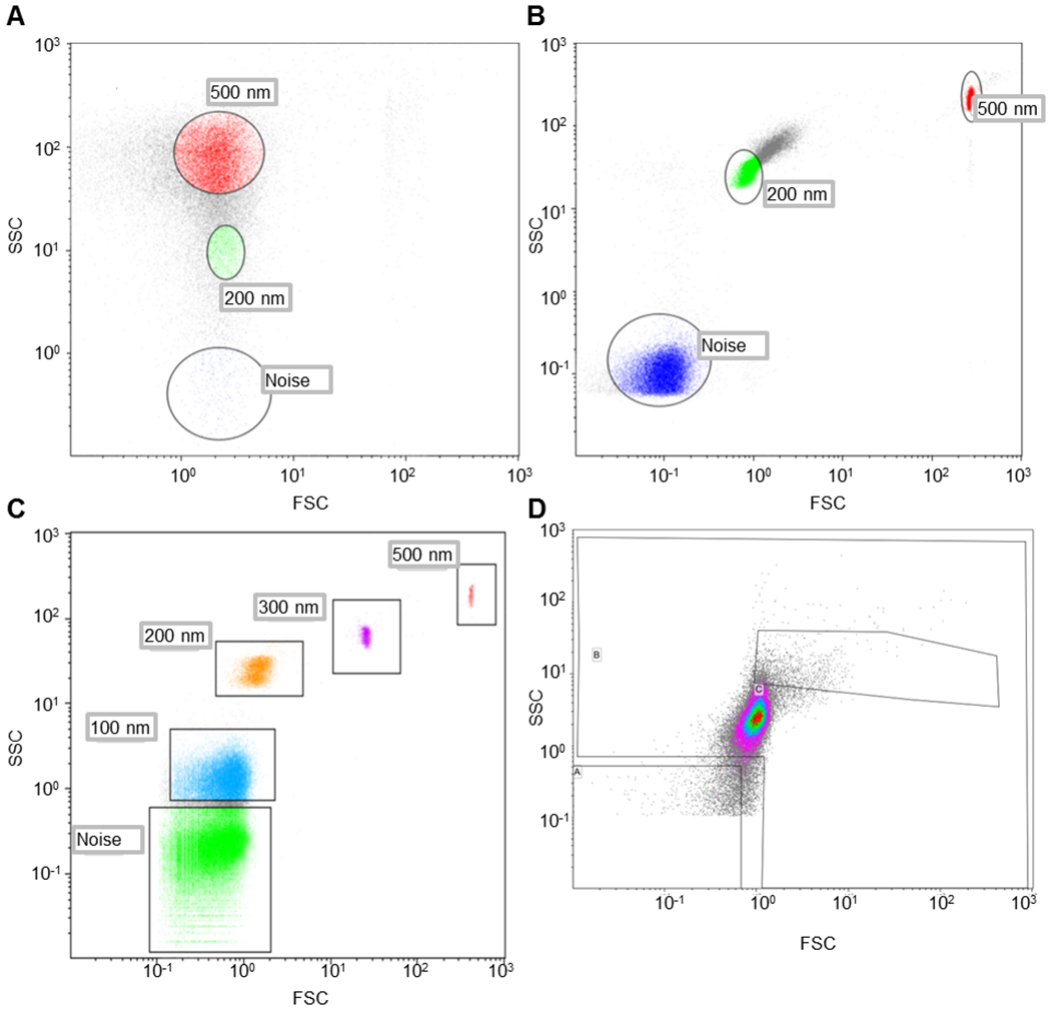
NanoFCM allows identification of beads and liposomes down to 100 nm. Separation of a mixture containing 200 and 500 nm latex beads by LSRII (A), and NanoView (B) instruments show more distinct separation with the NanoView Instrument. The NanoView is capable of separating a mixture of 100–500 nm beads into distinct populations (C) and can detect 100 nm liposomes (D). The gating strategy for these experiments to determine instrument and background noise are described in the methods section.

Fig 1 shows the limited separation of noise and 500 nm latex beads, with an undistinguishable 200 nm latex bead population when recorded with a standard LSRII machine (Fig 1A) compared to a significantly more distinct separation with the NanoView-equipped MoFlo XDP cell sorter (Fig 1B). Furthermore, nanoFCM can effectively identify and separate unique populations from a mixture of 100, 200, 300 and 500 nm beads (Fig 1C) based on side-scatter trigger. Due to the large differences in refractive index between PCS beads (1.59) and membrane bound EVs normally found in biological fluids (~1.36), it is difficult to estimate the actual size distribution of an EV population based on size calibration using latex or polyester bead. Therefore, we further validated the identification capabilities of the instrument using 100 nm liposomes. Fig 1D shows a population of 100 nm liposomes, demonstrating the ability of the NanoView to identity physiologically relevant nanovesicles of a similar size to EVs.

### Analysis of human plasma EVs by nanoFACS

**Fig 2:**
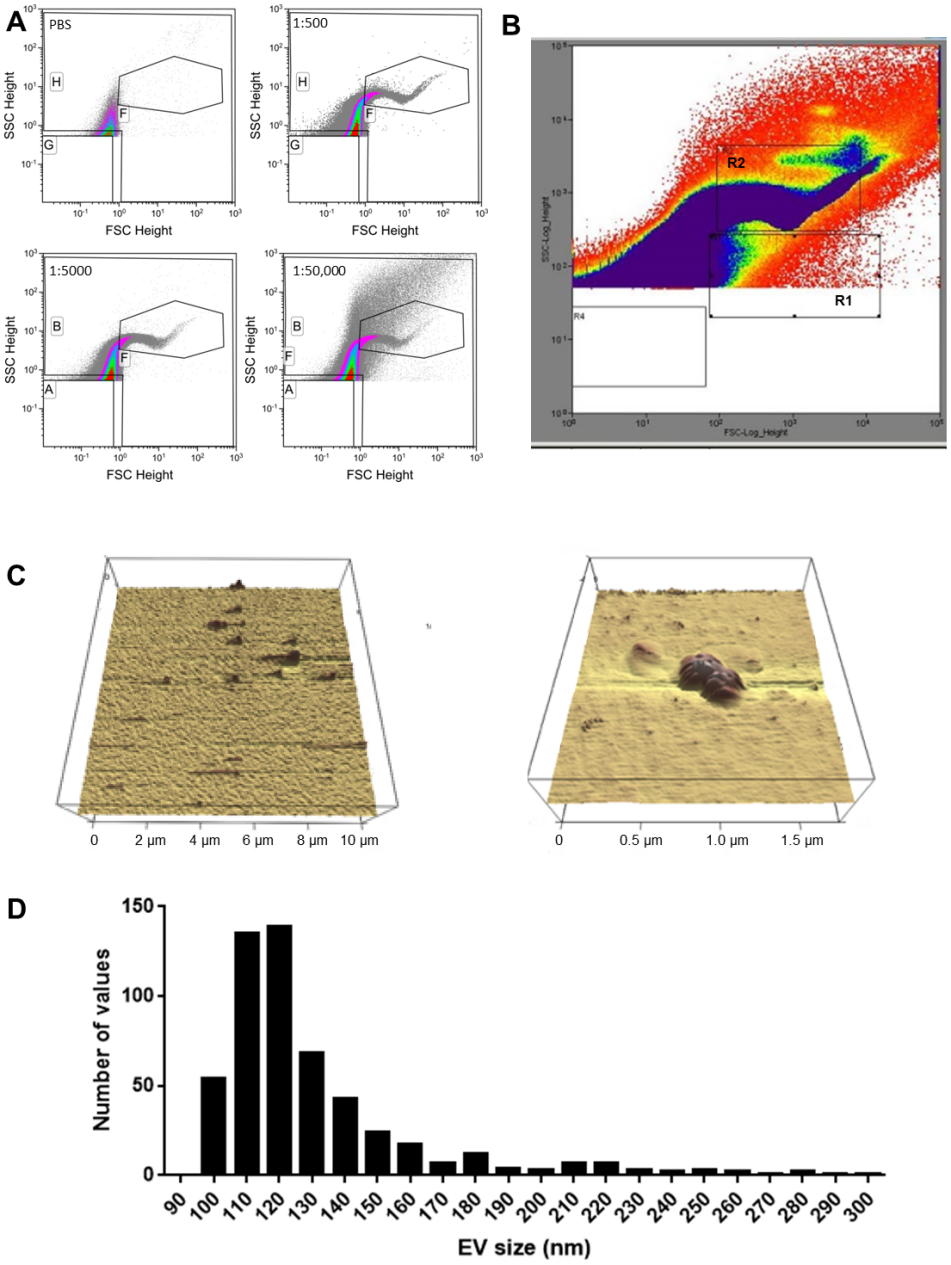
EVs detected in plasma from a healthy donor. Plasma from 5 mL of blood was collected, centrifuged to remove cellular debris (see methods), and imaged following a series of dilutions in PBS (A) to test for ‘swarming’. An EV population (based on positioning of 100 nm liposomes and size distribution of plasma samples) from the plasma of a healthy donor was sorted (gate R2; B) and imaged using atomic force microscopy (C). The size distribution of sorted EVs was analyzed by qNano and is represented in D. The gating strategy for these experiments is detailed in the methods section.

An important consideration in the analysis of submicrometer samples by flow cytometry is the possibility of ‘swarming’ [13] which is created when numerous EVs pass simultaneously through the interrogation area of the flow cytometer and are acquired as one large event. To determine whether swarming occurred with our experimental setup we analyzed cell free plasma from a single healthy donor diluted from 1:100 to 1:50,000 with PBS. Fig 2A shows 1:500, 1:5000, and 1:50,000 dilutions of plasma, which display a distinctive expression pattern of EVs that is not significantly altered across dilutions. As the dilution factor increased, mean acquisition time increased, while the total number of events acquired remained constant (Table 1). This confirms that the instrument’s operational parameters are sufficiently optimized to avoid the effect of swarming. Finally, to confirm the size of EVs detected by flow cytometry, a gated regions corresponding to the areas with expected sizes of 100–200 nm (based on bead and liposome sizing) were sorted (Fig 2B; gate R2) and imaged by AFM (Fig 2C). The size distribution of these sorted particles were further confirmed by tunable resistive pulse sensing using the qNano system (Izon). From a total of 535 individual EVs that were measured, the median size was 120 nm with a minimum of 96 nm and a maximum of 298 nm. A histogram of the size distribution of EVs is shown in Fig 2D.

**Table 1:** Percentage gated and number of events in a series of plasma dilutions from a single healthy donor.

Sample	% Gated	Number of events	Mean Acquisition Time (s)
**PBS**	99.93	30,079	
**Plasma 1:100**	99.84	499,214	8.18
**Plasma 1:500**	99.91	499,558	12
**Plasma 1:5000**	99.91	499,538	213.83
**Plasma 1:50,000**	98.94	494,718	367.44

### The distribution of EVs from healthy donors varies during the day

**Fig 3:**
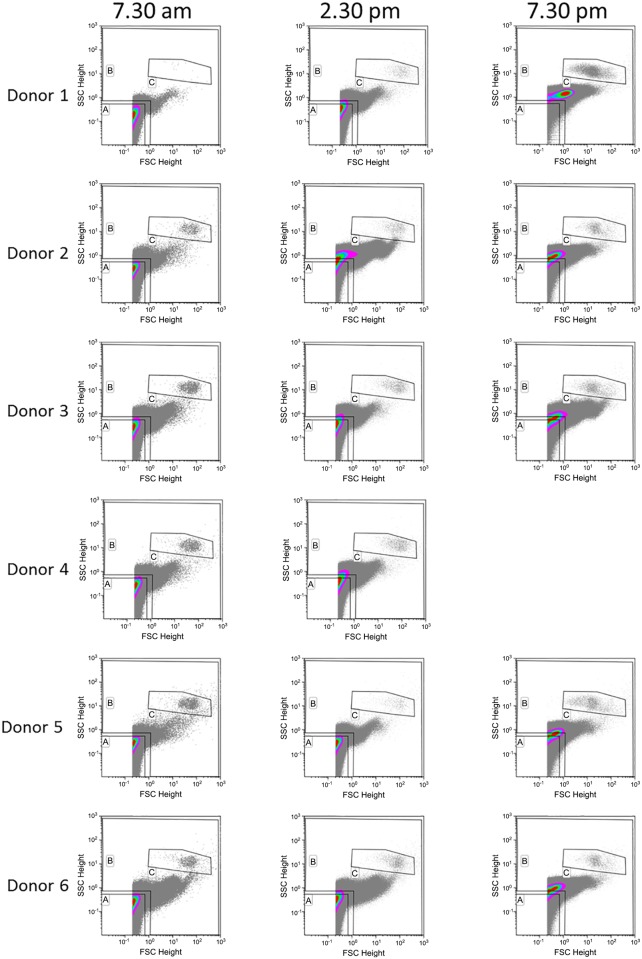
The number and size distribution of plasma EVs isolated from healthy donors varies during the day. Plasma from 6 healthy donors was isolated and cell-free plasma was diluted 1:500 in PBS and imaged using nanoFCM. Plots from all donors recorded at 7:30 AM, 2:30 PM, and 7:30 PM are shown.

Plasma from 6 healthy donors was collected, immediately processed, and analyzed over the course of one day at 7:30 AM, 2:30 PM, and 7:30 PM (Fig 3). The distribution of EVs detected by nanoFCM showed variance across different time points for all donors, as reflected by changes in Geo Means for both forward and side scatter (Fig 3, Table 2). We found variance in the relative size distribution of EVs: although not uniform, we observed a trend with the vesicles seen in the evening (and to some degree in the afternoon) being larger in size (based on scatter profile) compared to the ones observed in the morning. Interestingly, EVs collected in the evening had the largest range of sizes, containing EVs as large as those found during the afternoon alongside a peak population significantly smaller than morning EVs. Taken together these findings indicate that EVs in plasma of healthy individuals are dynamic structures, with their number and size distribution changing continuously.

**Table 2:** Geo Mean values for forward and side scatter from the 6 healthy donors at the 3 time points collected.

	Forward Scatter Geo Mean			Side Scatter Geo Mean		
Donor #	7.30 am	2.30 pm	7.30 pm	7.30 am	2.30 pm	7.30 pm
**1**	0.77	0.43	0.90	0.76	0.86	1.30
**2**	0.42	0.60	0.52	0.83	1.06	1.05
**3**	0.56	0.41	0.74	0.81	0.90	0.94
**4**	0.52	0.92		0.81	0.41	
**5**	0.49	0.54	0.56	0.84	0.83	0.90
**6**	0.86	0.65	0.51	0.74	0.84	1.00

## Discussion

Detection of EVs requires instrumentation capable of reliable identification of particles down to 100 nm in samples that undergo minimal processing. Commercially available flow cytometers equipped with optics up to 0.65 NA are usually able to identify particles (“events”) down to a size of about 500 nm, which is significantly larger than most plasma EVs. Previous studies have used fluorescence-based thresholding to detect labeled beads or immune cell-derived EVs in the 100–200 nm range effectively [14]. While effective, this method relies on labeling of the EVs with fluorescent dye PKH67 or with fluorescent-conjugated antibodies [15, 16].

Roussea and colleagues also used YFP-CD41 transgenic mice, which express YFP in platelet-microparticles and provided an ‘*in vivo’* labeled population of platelet-derived EVs. While these techniques have their advantages, they also require initial isolation of EVs using centrifugation or other methods (for labeling), which introduces variables and biases. Secondly, there are no known ‘universal’ antigens for EVs, hence an antibody-based labeling of EVs would not be suitable for unbiased profiling of EVs directly from human subjects, as we have shown in this manuscript. Finally, the use of dyes to label EVs requires careful washing to prevent artifactual signal from dye aggregates. Our study is complementary to these studies and uniquely suited to the answer the questions we sough to answer.

In this study, we have demonstrated that the NanoView integrated MoFlo XDP cell sorter is capable of detecting latex beads, liposomes, and human plasma EVs down to 100 nm. Furthermore, this instrumentation is capable of providing information on the relative size and distribution of EVs in human plasma. Along with other recently described methodologies for assessing EV profiles in a relatively unperturbed state [17], the ‘nanoFCM’ methodology described here will provide critical information in accurately characterizing variations in relative EV sizes and counts. Such information would be critically important in interpreting changes in EVs with disease.

EVs have only recently emerged as potential biomarkers and cell-cell communicators and could be utilized as a valuable tool in diagnostics, prognostics, and disease treatment. However, the technology available for EV detection and evaluation needs further advancement. Flow cytometry provides greater potential in screening and quantifying EVs because of its ability to quantitate large numbers of events and characterize them by both physical and fluorescent properties. The analysis of EVs using flow cytometry has several significant limitations, stemming mostly from their size and low scattering properties. When using forward- and side-scattering triggers, standard flow cytometers allow for accurate identification and measurement of free floating particles that are approximately 500 nm in diameter and above [18]. Thus, most of the EVs present at any given time in plasma and other biological fluids are not able to be resolved as single particles above the limit of detection in conventional flow cytometers. While various plastic, ‘size’ bead standards currently available could provide an accurate and easily obtainable size reference for EVs, their light-scattering properties vary drastically from those of EVs, and therefore may not provide a very accurate estimate of EV sizes [19]. This study demonstrates that it is possible to detect EVs in the range of 100 nm, and that nanoFMC is a valuable tool for visualizing the distribution of EVs in the 100–500 nm size range, and how those EV distribution vary during the day. Further refinement of this instrumentation is needed for resolution and analysis of EVs smaller than 100nm, and because nanoFCM only provides a relative assessment of size (light scattering properties) and quantity of EVs; thus, further refinement of instrumentation and methods are required for quantitative sizing and counting of EVs.

We have shown that the relative quantity and size of EVs found in blood drawn from healthy donors varies during the day. While the observed diurnal variations were not entirely consistent between subjects, every subject showed some level of diurnal variation. The number of EVs in blood at a given time is the result of a delicate balance between production and uptake of EVs by various tissues and organs, and could be influenced by multiple factors including exercise [20], time since last meal, gender, and circadian variations [21]. There is a current lack of information on the normal physiology of EVs in health, which will only be remedied by further, more in-depth studies involving a larger number of patients. However, this pilot study demonstrates the phenomenon of diurnal variation in EVs. This is of paramount importance when considering the use of EVs as potential biomarkers in human health and disease.

The data that we present here demonstrate the feasibility of identifying and discriminating EVs in human plasma down to a size of 100 nm by flow cytometric methods, and the use of these methods to identify and study diurnal variations in circulating plasma EVs.

## Supporting Information

S1 Fig Shedding of debris from commonly used copolymer tubes. PBS from the same batch was placed in microcentrigue tubes (USA Scientific, 1415–2500; **A**) or quick-seal tubes (Beckman Coulter, 342184; **B**) prior to flow cytometry analysis. Beckman Coulter tubes were found to shed debris within the size and scatter range of EVs and were not used for further experiments.
(TIFF)

S2 Fig The MoFlo XDP standard FSC photodiode and SSC PMT are compared to the NanoView FSC PMT and optical enhancements. The NanoView module shows enhanced dynamic range, better signal to noise ratio and overall better small particle detection based on nanoparticle sizing.
(TIFF)
