# Supplementary figures and images for: Diurnal Variations of Circulating Extracellular Vesicles Measured by Nano Flow Cytometry
Source: PLoS One. 2016 Jan 8;11(1):e0144678. doi: 10.1371/journal.pone.0144678 (PMC4706300; doi:10.1371/journal.pone.0144678)

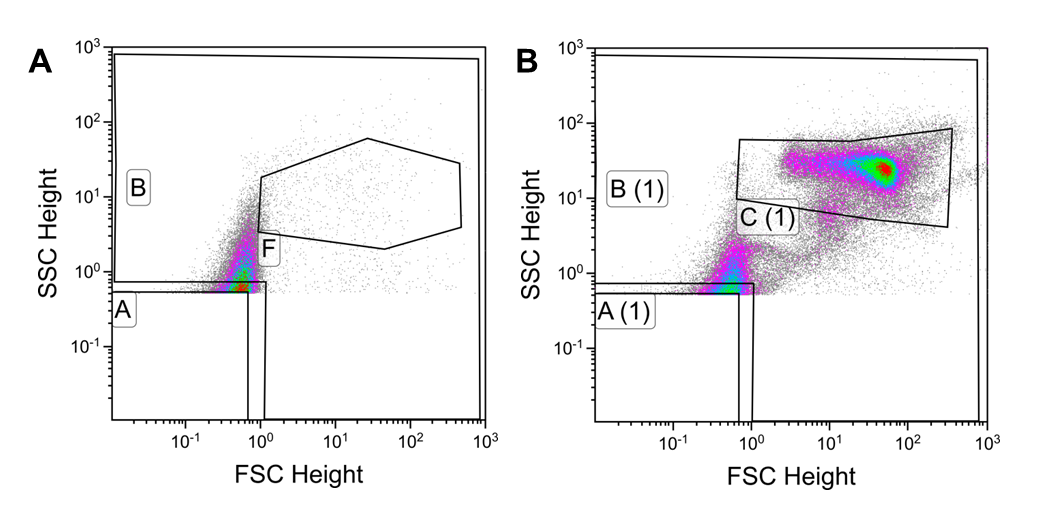

Supplement: S1 Fig — PBS from the same batch was placed in microcentrigue tubes (USA Scientific, 1415–2500; A) or quick-seal tubes (Beckman Coulter, 342184; B) prior to flow cytometry analysis. Beckman Coulter tubes were found to shed debris within the size and scatter range of EVs and were not used for further experiments. (TIFF) [file pone.0144678.s001.tiff]

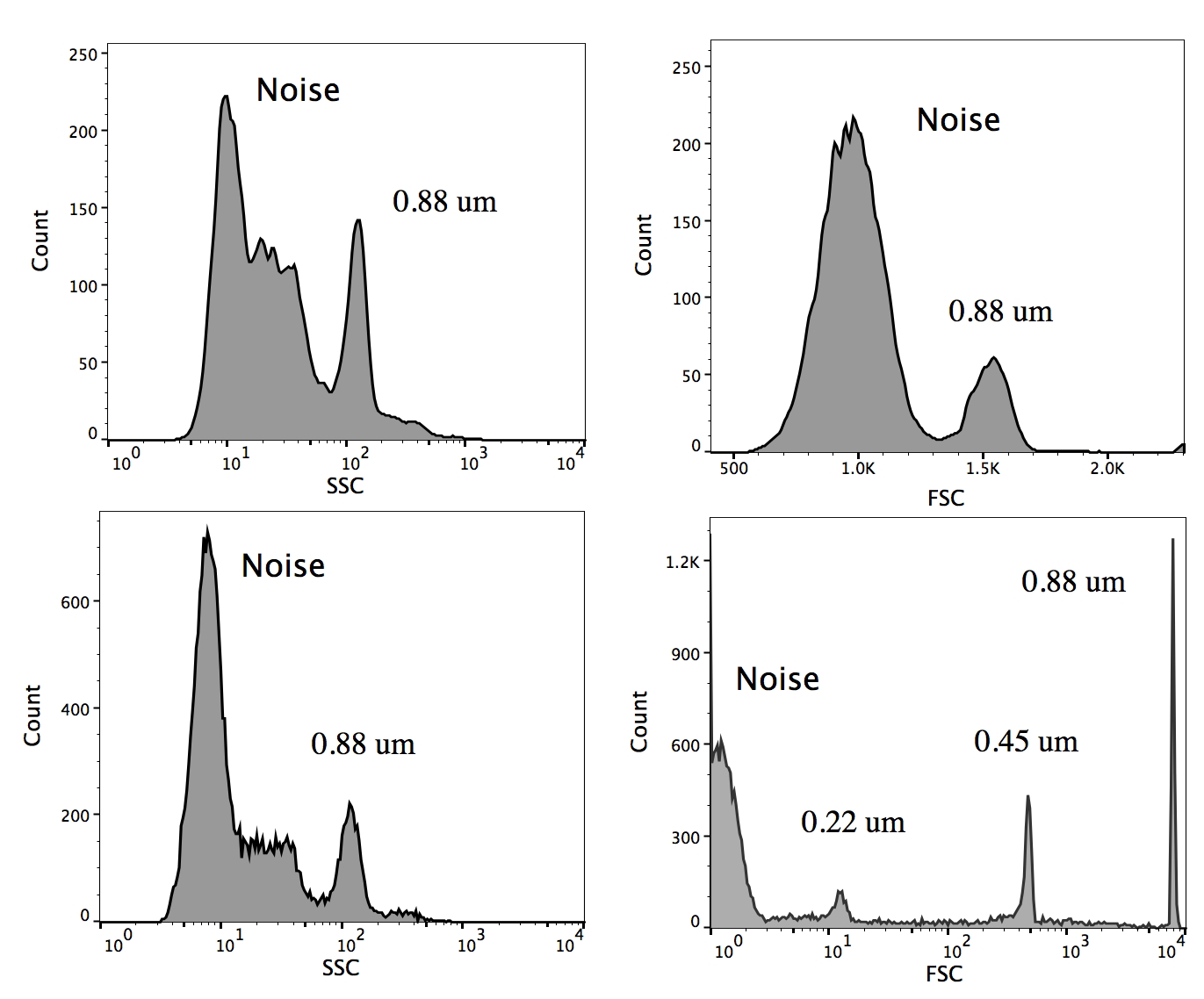

Supplement: S2 Fig — The NanoView module shows enhanced dynamic range, better signal to noise ratio and overall better small particle detection based on nanoparticle sizing. (TIFF) [file pone.0144678.s002.tiff]
